# Effects of Non-Pharmacological Interventions on the Biopsychosocial Health of Community-Dwelling Older Adults with Chronic Heart Failure: An Integrative Review

**DOI:** 10.3390/healthcare14131997

**Published:** 2026-07-05

**Authors:** Miguel Gerez-De-Paco, Dulcenombre de María García-López, Anabel Chica-Pérez, Cayetano Fernández-Sola, Adrián Martínez-Ortigosa, María del Mar Jiménez-Lasserrotte

**Affiliations:** 1 Primary Care Services, Levante-Alto Almanzora Health District, 04600 Almeria, Spain; mgd461@ual.es; 2 Hospital Universitario Torrecárdenas, 04008 Almeria, Spain; dgl896@inlumine.ual.es; 3 Department of Nursing, Physiotherapy and Medicine, University of Almeria, 04120 Almeria, Spain; cfernan@ual.es (C.F.-S.); mjl095@ual.es (M.d.M.J.-L.); 4 Facultad de Ciencias de la Salud, Universidad Autónoma de Chile, Temuco 4810101, Chile; 5 Faculty of Nursing and Podiatry, Department of Nursing, University of Valencia, 46010 Valencia, Spain; adrian.martinez-ortigosa@uv.es

**Keywords:** chronic heart failure, community dwelling, nursing, older adults, non-pharmacological interventions, integrative review

## Abstract

**Background/Objectives**: Chronic heart failure (CHF) is a leading cause of global morbidity and mortality, particularly among older adults, significantly impacting their quality of life and imposing a substantial economic burden. While pharmacological and surgical treatments remain essential, non-pharmacological interventions led by nurses are gaining prominence due to their comprehensive approach and biopsychosocial impact. The objective of this study was to synthesise and integrate such interventions for community-dwelling older adults with CHF. **Methods**: An integrative review was conducted in accordance with the Joanna Briggs Institute protocols and the PRISMA statement, utilising a systematic search across databases including PubMed and Cochrane. Qualitative, quantitative, and mixed-methods studies evaluating non-pharmacological interventions in the home setting were included, whilst those targeting non-specific populations were excluded. Following a rigorous screening process, 12 studies were selected, and their methodological quality was appraised based on study design. **Results**: The 12 included studies involved a total of 2466 participants and addressed interventions across the domains of education, physical activity, telehealth, and nutrition, with programme durations ranging from 4 weeks to 16 months. Notable improvements were observed in physical capacity, cognitive function, quality of life, and self-care capabilities, alongside potential reductions in hospitalisations reported in some studies. However, considerable methodological variability was identified across the literature. **Conclusions**: This review synthesises non-pharmacological nursing interventions for older adults with CHF, demonstrating varied benefits across multiple biopsychosocial domains. The findings emphasise the critical need for further research to evaluate the economic viability of these programmes and to adapt interventions to enhance the delivery of community-based care.

## 1. Introduction

Chronic heart failure (CHF) accounts for approximately 36% of all cardiovascular disease deaths worldwide [1]. It is estimated that CHF affects around 64.3 million people globally [2], with the prevalence in European countries reaching almost 2% of the adult population [3]. Older adults aged over 60 represent 80% of those affected [4]. CHF significantly affects those who suffer from it, encompassing a wide range of symptoms from the mildest, such as occasional fatigue and mild dyspnoea, to others like pulmonary congestion and thrombosis [5]. These symptoms ultimately impair their quality of life [6] and reduce their capacity to perform basic activities of daily living [7]. In most cases, CHF gives rise to other pathologies such as arterial hypertension, dyslipidaemia, atrial fibrillation, and renal failure [3]. Furthermore, it is estimated that because of this, global healthcare systems allocate enormous financial resources annually to combat CHF amongst older adults [8]. Consequently, it is necessary to develop and refine various non-pharmacological interventions focused on the treatment [9] of this condition in older patients within the community setting.

CHF is a chronic cardiovascular condition characterised by the damage or loss of myocardial cells, increased haemodynamic stress, and structural impairment, with clinical manifestations that include dyspnoea and fluid retention [10]. CHF in older adults has been associated with a decline in functional capacity [11] and increased frailty [12]. Available evidence suggests that CHF is associated with anxiety, depression [13], stress, and cognitive impairment [14]. Furthermore, emerging evidence suggests that emotional dysregulation and affective disturbances may influence both psychological well-being and physical health outcomes due to complex mind–body interactions. This can potentially affect symptom perception, self-care behaviours, and quality of life in people living with chronic conditions [15]. Moreover, CHF is also linked to unwanted loneliness and a loss of social support in older adults [16], which can aggravate the symptoms of the disease and contribute to increasing associated healthcare expenditure [17,18].

In general, first-line therapy for CHF focuses on pharmacological treatment [19]. However, these treatments are not fully effective in relieving CHF symptoms [20,21]. Furthermore, several sources state that the prolonged use of some of the drugs used could be associated with adverse effects that affect the quality of life and functionality of older adults [22,23]. Alternatively, various surgical interventions have been implemented, such as cardiac ablation, valve repair, and coronary artery bypass grafts [24,25]. Nevertheless, surgery is reserved for advanced stages of the disease [26] and is associated with potential risks, such as nosocomial infections and immunological reactions [27]. For this reason, nurses face the challenge of implementing and evaluating non-pharmacological interventions that contribute to improving the situation of older patients with CHF at a biopsychosocial level.

Nurses are the frontline of care for older adults in the community setting [28]. Therefore, it is crucial to know what interventions nurses can implement in this context to improve CHF in older adults [29]. To date, several systematic reviews have been conducted to evaluate the effectiveness of drug use in the treatment of chronic heart failure in older adults [30,31,32]. Literature reviews have also been carried out to evaluate the effectiveness of surgical interventions [33], as well as advanced therapies, such as ventricular assist devices [34]. Reviews of non-pharmacological interventions in patients with CHF have also been conducted [35,36,37]. Several of these reviews do not focus on older adults [38,39] or are not exclusive to the community setting [40]. Thus, the general objective of this review is to synthesise and integrate the available evidence on non-pharmacological nursing interventions implemented and evaluated to improve biopsychosocial factors in older adults with CHF living in the community setting. Furthermore, this work had the following specific objectives: (1) To identify what type of nursing interventions have been applied and evaluated to improve biopsychosocial factors in older adults with CHF; (2) To describe the characteristics (duration, frequency) of these interventions; (3) To evaluate the outcomes reflected in the studies that have implemented and evaluated these interventions. Given the heterogeneity of non-pharmacological interventions and outcome domains in chronic heart failure, an integrative review was deemed the most appropriate approach.

## 2. Materials and Methods

### 2.1. Design

This integrative review follows the Joanna Briggs Institute (JBI) methodology for integrative reviews [41] and complies with the Preferred Reporting Items for Systematic Reviews and Meta-Analyses (PRISMA) checklist [42]. The protocol for this study was registered with INPLASY as an integrative review (Registration number: INPLASY202560052, https://doi.org/10.37766/inplasy2025.6.0052 (accessed on 20 May 2026)). This approach enables a rigorous and comprehensive synthesis of heterogeneous evidence, which is particularly relevant in the context of multifaceted nursing interventions.

### 2.2. Search Strategy

To select the studies, a search was conducted between November 2024 and January 2025 using a three-phase process. In the first phase, an initial search was performed in the following databases: MEDLINE (via PubMed), Cochrane, CINAHL, Web of Science, SCOPUS, and Google Scholar. Google Scholar was searched as a complementary source of grey literature rather than as a primary indexed database, given its known reproducibility limitations. During this phase, keywords for the titles and abstracts of the articles were identified, including terms such as “aged”, “Congestive Heart Failure”, “Community Dwelling”, “Intervention”, “Non-pharmacological”, and variations related to these terms, which are presented in Table A1 of Appendix A. In the second phase, the natural language from the first phase was combined with MeSH terms associated with the topics, such as “aged”, “Living at home”, and “Cardiac Failure”. For this purpose, the Boolean operators “OR” and “AND” were used, and a search strategy was created. In the third phase, the search strategy was adapted for each database, with the one used in PubMed being as follows: ((aged) OR (Elderly) OR (Older adults) OR (Older)) AND ((Congestive Heart Failure) OR (Cardiac Failure) OR (Heart Failure, Congestive) OR (Myocardial Failure)) AND ((Living, Independent) OR (Aging in Place) OR (Community Dwelling) OR (Dwelling, Community) OR (Dwellings, Community) OR (Home Environment*) OR (Living at home)) AND ((Patient compliance) OR (Patient Cooperation) OR (Patient Adherence) OR (Treatment Compliance) OR (Therapeutic Compliances) OR (Client Adherence) OR (Client Compliance) OR (Exercise) OR (Physical Exercise) OR (Physical Activity) OR (Exercise Training) OR (Exercises) OR (Behavioral) OR (Behaviors) OR (Cognitive Behavioral Therapy) OR (Behavioral Therapy, Cognitive) OR (Cognitive Behavioral Therapies) OR (Therapies, Cognitive Behavior) OR (Cognition Therapy) OR (Cognitive Therapy) OR (Cognitiv*) OR (Therapy, Cognitive) OR (nutrition) OR (Nutr*) OR (Nutrition Policies) OR (Food Policy) OR (Dietary Guideline*) OR (Guideline, Dietary) OR (Nutrition Guideline*) OR (Nutraceutical) OR (Non-pharmacological) OR (Rehabilitation) OR (Rehab*)) AND ((Intervention*) OR (Program*) OR (Programme*)). The strategies adapted for each database can be consulted in Table A2 of Appendix A.

### 2.3. Eligibility Criteria

Eligibility criteria were established to reflect the objectives of the review, specifying the population, the interventions of interest, and the context in which they were implemented.

- Participants: studies involving cohorts of older adults (>60 years) with CHF.
- Concept: studies examining non-pharmacological interventions, including physical exercise, nutrition, and behaviour change, aimed at improving biopsychosocial outcomes in older adults with chronic heart failure (CHF).
- Context: studies of interventions implemented and evaluated in the home setting.

This review included qualitative, quantitative, and mixed-methods studies. Articles in English, Spanish, French, and Portuguese were accepted for inclusion. Studies of interventions not executed exclusively in the community setting or not involving older adults were excluded. Studies of non-pharmacological interventions in older adults residing in the community setting with cardiovascular disease who did not present specific results sections for CHF were excluded. Studies with publication dates older than 10 years were excluded.

### 2.4. Article Selection and Data Extraction

**Figure 1:**
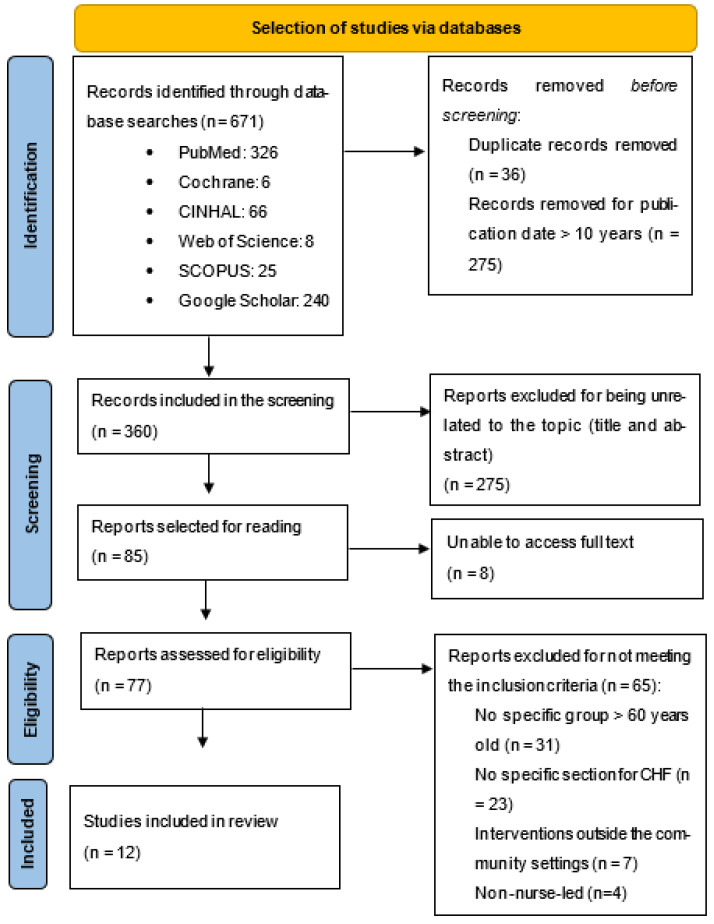
Flowchart of the study selection process according to PRISMA 2020 [42].

After applying the search strategies to the aforementioned databases, the following number of articles were obtained: PubMed (*n* = 326), Cochrane (*n* = 6), CINAHL (*n* = 66), Web of Science (*n* = 8), SCOPUS (*n* = 25), and Google Scholar (240), amounting to a total of 671 articles. During the identification phase, articles older than 10 years (*n* = 275) and duplicates (*n* = 36) were removed. In the screening phase, unrelated articles were removed after reading the title and/or abstract (*n* = 275). In the eligibility phase, two reviewers from the research team reviewed 85 articles. Those articles that could not be retrieved (*n* = 8) were excluded, along with 65 that did not meet the inclusion criteria. Finally, 12 articles were included in the review. Study selection was performed independently by two reviewers. Disagreements were resolved through discussion until consensus was reached. The selection process is reflected in the flow diagram (Figure 1).

### 2.5. Data Extraction and Synthesis

Two researchers extracted information from the studies included in the review. The methodology proposed by the JBI was used, taking into account the objectives and research questions of this review. A data table with the following categories was used: author, domain, design, population, objective, person responsible for the intervention, intervention, evaluations and follow-up, outcome measures, and results. The data extraction methodology was piloted with one of the selected articles. The reviewers found no discrepancies in the extracted data nor detected any problems in the process. Consequently, the reviewers used this methodology to extract information from all articles. Finally, a third reviewer confirmed the accuracy and integrity of the extracted data. A summary of the extracted information is presented in Table 1.

### 2.6. Critical Appraisal

Methodological quality of included studies was critically appraised using JBI Critical Appraisal of Evidence Synthesis tools [43,44,45]. Two reviewers independently conducted critical appraisal of the studies, which were subsequently reviewed by the most experienced researcher included in the study. To this end, 4 tables were created in which the selected articles were included according to their design. One table was designed to assess the quality of randomised controlled trials (RCTs), another for quasi-experimental studies, one for cohort studies, and another for qualitative studies. The columns of each table correspond to the items to be evaluated according to the respective tools for each type of article. Subsequently, a quality score was obtained for each study (Table 2, Table 3, Table 4 and Table 5).

**Table 1:** Research articles included in this integrative review.

Author (Year)	Domain	Design	Population	Objective	Responsible for Intervention	Intervention	Measurement and Follow-Up	Outcome Measures	Main Results
(Dickson et al., 2014) [46]	Education/Cognitive	RCT	75 patients with CHF, mean age (69.9 ± 10 years), (53% female).	To assess the effectiveness of a community-based intervention aimed at developing skills related to self-care, knowledge, and HRQoL.	Health educators	IG: 2 bi-weekly sessions of 60 min/week × 4 weeks CG: usual care	Pretest 1 month 3 months	Self-care capacity: SCHFI V6.2 Knowledge of CHF: DHFKS HRQL: KCCQ CHF degree: NYHA class	Improved self-care capacity in IG vs. CG; (66.8 (18)) vs. (49.4 (21)); SCHFI = (Cohen’s f = 0.45; *p* = 0.02) Improved knowledge of CHF in IG vs. CG; (12.7 (1)) vs. (10.1 (3)); DHFKS = (Cohen’s f = 0.54; *p* = 0.001).
(Radhakrishnan et al., 2016) [47]	Education/Cognitive	Quasi-experimental	19 patients (14 of them over 60 years old); (data from F7, F9, F13, F18, and F20 removed).	To develop and test the prototype of a digital game focused on improving self-management knowledge and skills in older adults with CHF.	Nurse	Prior use: 45 min with researchers. At home: 30 min per session, 3 times a week. Functional evaluation and daily log for 4 weeks.	Pretest Post-test: 4 weeks	Knowledge of CHF: AHFKT Self-care capacity: SCHFI Intrinsic motivation: Subscale Of Intrinsic Motivation Inventory	Significant increase in disease knowledge at 4 weeks (22.26 vs. 25.00; *p* = 0.007) No significant differences found in self-care capabilities via the SCHFI. Patients reported the system was interesting, enjoyable, and easy to use.
(Kalter-Leibovici et al., 2017) [48]	Education + Telehealth	RCT	1360 older adults with CHF	To determine the effectiveness of a multidisciplinary disease management program	Nurse + Cardiologist + Nutritionist	Disease management: weekly remote contact via phone/video + centre visits × 6 months Usual Care: cardiology visits	Pretest 6 months post-test 12 months post-test 18 months post-test 24 months post-test	Time To First Readmission: years CHF stage: NYHA class Quality of life: SF-36 Depression severity: PHQ-9	Longer time to readmission IG vs. CG; (Mean = 3.0 years; range = 2.3–3.8 years) vs. (Mean = 2.2 years; range = 1.8–2.8 years) No difference in 6MWT; IG = lower risk of depressive symptoms.
(Antonicelli et al., 2016) [49]	Physical Activity	RCT	343 patients over 70 years old with a mean age of 76.90 ± 5.67	To determine the effect of an exercise protocol on functional capacity, hospitalisation, and quality of life in patients with CHF over 70 years of age.	Nurse + Cardiologist + Physiotherapist	TCG: Phase 1: 3 sessions per week for 3 months. Phase 2: remote nursing sessions monitoring symptoms for 3 months. UCG: PC visits and 2 cardiology visits over 12 months.	Pretest 3 months 6 months	Quality of life: MLHFQ Functional capacity + ADL + IADL + 6MWT: INTER-RAI-HC NT-proBNP: PG/ML Readmissions: per unit	Improved results in 6MWT at 3 months TCG vs. UCG; (380.7 ± 120.3) vs. (300.6 ± 125.7) and at 6 months; (394.1 ± 123.6) vs. (301.2 ± 125.8); *p* < 0.001. Less readmissions TCG vs. UCG; (15.2%) vs. (36.8%); *p* < 0.001 Improved quality of life TCG vs. UCG at 6 months; (28.6 ± 12.3) vs. (44.5 ± 12.3); MLHFQ = *p* < 0.001 Decreased NT-proBNP at 6 months; (440 (208)) vs. 2143 (1638)); pg/mL = *p* < 0.001
(Yeh et al., 2016) [50]	Physical Activity	Qualitative	100 older adults with CHF associated with centres in Boston.	To explore the physical and psychological effects, alongside the complete experience perceived by the patient, following a Tai Chi programme.	Nurse + Tai-Chi Instructor	TC group: 1 h sessions, twice a week/free practice at home, twice a week. Education group: basic education, twice a week.	12 weeks post intervention	Improvement in functional and psychological aspects: Interview	Participants in the Tai Chi group significantly increased their level of moderate physical activity upon completion. Qualitative findings showed greater overall relaxation, stress alleviation, enhanced resilience with reduced emotional automaticity, and a newfound connection with bodily sensations and symptoms.
(Dibben et al., 2023) [51]	Physical Activity	RCT	247 older adults with CHF	To evaluate the impact of home-based cardiac rehabilitation on the physical activity of heart failure patients and to explore the factors that influence changes in activity.	Nurse + Physical Therapy + Occupational Therapy	REACH-HF: Participants received a self-management program with a manual, and exercise was recommended 3 or more times per week. CG: Usual care.	Pretest 4 months 6 months 12 months	Quality of life: -MLHFQ -EQ-5D-5L Physical activity: GENEActiv accelerometer	Greater light activity REACH-HF vs. Control: (8.12 ± 87.01) vs. (−10.43 ± 87.85); (t = 1.28; *p* = 0.20) Less inactivity REACH-HF vs. Control: (−12.12 ± 115.83) vs. (15.53 ± 110.52); (t = −1.51; *p* = 0.13)
(Gholami et al., 2024) [52]	Physical Activity	RCT	75 older adults with CHF.	To determine the effect of a supervised balance training program on cognitive function and ADLs.	Nurse	BT: 4 weekly sessions—30 min/session—8 weeks UC: cardiologist visits—no specific exercise	Pretest Post-test—8 weeks	HF degree: NYHA class Cognitive function: MoCA-B ADL: Barthel Index IADL: Lawton Body Mass: BMI	Higher cognitive function BT vs. UC; (18.33 ± 4.88) vs. (14.71 ± 4.23); MoCA-B = (t = 0.167; *p* < 0.001) Improvement in IADL BT vs. UC; (5.83 ± 1.46) vs. (4.46 ± 2.15); Lawton = (t = 0.705; *p* < 0.001) Improvement in ADL BT vs. UC; (97.80 ± 15.09) vs. (88.89 ± 9.37); Barthel = (t = 0.379; *p* < 0.001).
(Donesky et al., 2017) [53]	Physical Activity + Telehealth	Quasi-experimental	14 older adults	To assess the feasibility and clinical outcomes of a video-call-delivered yoga programme in subjects with COPD and CHF simultaneously.	Nurse + Yoga Instructor	Tele-yoga group: 2 classes × week for 8 weeks. Control group: educational material via post, once a week for 8 weeks + 15 min nursing call/week.	Pretest 8-week post-test	Strength: -Bicep curls in 30 s -Sit-Stands in 30 s Cardiovascular fitness: 6MWT Quality of life: KCCQ Depression: (PHQ)-8 Dyspnoea: Dysnea-12 + Borg Scale Insomnia: General Sleep Disturbance Scale	Dyspnoea during 6MWT improved in IG vs. CG; (0.5 (0–2.7)) vs. (2.4 (0.2–4.6)); *p* = 0.03; effect size = −1.27. Breathing difficulty during 6MWT improved in IG vs. CG; (2.8 (0–5.6)) vs. (4.3 (1.6–7.1)); *p* = 0.02; effect size = −1.402. Depression symptoms decreased in IG and increased in CG; (7.2 (1.3–13.0)) vs. (8.6 (3.6–13.7)); *p* = 0.48; effect size = −0.385. Insomnia improved in IG vs. CG; (40.5 (17.1–63.9)) vs. (62.8 (41.7–83.9)); *p* = 0.09; effect size = −0.937. No differences in QoL between both groups.
(Lind et al., 2016) [54]	Telehealth	Cohort study	14 older adults with CHF	To report on the experiences of a pilot study based on the implementation of a home telehealth system for patients with HF.	Nurse	Daily recording of vital signs and symptoms; questionnaires regarding the system at 2–4 weeks.	Pretest 2–4 weeks post-test 1-year Follow-up	System usage testimonies: Likert-type scales Daily vital signs and symptoms: -Body Weight: kg -Blood Pressure: Mmhg -Dyspnoea: nº episodes Readmissions: per unit	Participants reported that the system was simple to manipulate and use. All patients successfully provided daily vital signs and symptom records. No patients experienced hospital readmissions during the study.
(Gokalp et al., 2018) [55]	Telehealth	Cohort	36 older adults	To investigate the potential of an integrated care system that acquires clinical sign data and vital habits to support independent living in older adults with chronic conditions.	Nurse	Daily monitoring of: -Ambulation -Time spent in rooms -BP -Weight	Continuous monitoring up to max. 495 days	Adherence: total days of use	BP measurement adherence in 60% of subjects. Total acceptance of monitoring in 85% of patients. Increase in specialized care and diagnoses thanks to the systems. Information successfully correlated with acute exacerbations, enabling action
(Nouryan et al., 2019) [56]	Telehealth	RCT	89 older adults	To compare emergency department visits, hospitalizations, length of stay, quality of life, and costs between the groups during the 6-month period.	Nurse + Cardiologist	HTM group: virtual telematics consultation once a week + daily monitoring of vital signs. COM group: usual care by cardiology.	Pretest 6 months	QoL: MLHFQ Emergency department visits: Units Hospitalizations: Units Cost: Dollars	Higher emergency department visits in COM vs. HTM; (60%) vs. (38%); *p* = 0.004 No differences in hospitalizations; QoL increases in both, especially HTM vs. COM; (−9.6) vs. (−3.6); MLHFQ = (*p* = 0.02).
(Persson et al., 2020) [57]	Telehealth	Cohorts	94 subjects (58 with CHF) aged between 65 and 100 years.	To evaluate whether the Health Diary telemonitoring system combined with hospital-based home care improves health-related quality of life in older adults with COPD and/or CHF and reduces hospitalizations and healthcare costs.	Nurse + Physician	Daily logging of symptoms and vital signs until the end of the study + contact with primary care on demand	Pretest 1 month 6 months 12 months	Health-Related Quality of Life: EQ-5D and RAND-36 Disease-specific quality of life: MLHFQ Healthcare system dependency: -no. Of hospitalizations -Contacts -Care costs	RAND-36 improvements in: -Physical functioning (RP) (+11.6 points at 1 month, +11.8 points at 6 months) -Social functioning (SF) (+10.3 points at 1 month, +11.0 points at 6 months, +11.2 points at 12 months) -Mental health (MH) (+7.1 points at 1 month, +8.8 points at 6 months) MLHFQ: overall improvement with a 6.4-point decrease at 1 month, highlighting the emotional domain Hospitalizations reduced by 74% compared to the previous year

6MWT (Six-Minute Walk Test). ADL (Activities of Daily Living). AHFKT (Atlanta Heart Failure Knowledge Test). BP (Blood Pressure). BT (Balance Training). BMI (Body Mass Index). CHF (Chronic Heart Failure). COM (comprehensive outpatient management). DHFKS (Dutch HF Knowledge Scale). COPD (Chronic Obstructive Pulmonary Disease). HRQL (Health-related quality of life). HTM (Home Telemonitoring). IADL (Instrumental Activities of Daily Living). INTER-RAI-HC (InterRAI Home Care Assessment System). KCCQ (Kansas City Cardiomyopathy Questionnaire). MLHFQ (Minnesota Living with Heart Failure Questionnaire). MoCA-B (Montreal Cognitive Assessment). NYHA (New York Heart Association). (PHQ)-8 (Personal Health Questionnaire). PHQ-9 (Patient Health depression scale). QoL (Quality of life). SCHFI (Self-Care of Heart Failure Index). SF-36 (Short Form 36 item test). TC (Tai-chi). TCG (Training care group). UC (Usual Care). UCG (Usual Care Group).

**Table 2:** Summary of the critical appraisal of the methodology of the randomised clinical trials included in the review (*n* = 9).

	Randomisation in Participant Allocation	Allocation Concealment	Similarity Between Control Group/Intervention Group	Blinding of Participants	Blinding of Administrators	Blinding of Outcome Assessors	Same Treatment Among Groups	Adequate Description and Analysis of Group	Participant Analysis in Assigned Groups	Same Measurements	Reliable Measurement of Results	Appropriate Statistical Analysis	Appropriate Trial Design	Total Quality Score
Dickson et al. (2014) [46]	Yes	Yes	No	No	No	Yes	Yes	Yes	Yes	Yes	Yes	Yes	Yes	10/13
Kalter-Leibovici et al. (2017) [48]	Yes	Yes	Yes	No	No	No	Yes	Yes	Yes	Yes	Yes	Yes	Yes	10/13
Antoniocelli et al. (2016) [49]	Yes	Yes	Yes	No	No	N/A	N/A	Yes	N/A	Yes	Yes	Yes	Yes	9/13
Dibben et al. (2023) [51]	Yes	N/A	Yes	No	No	N/A	Yes	Yes	Yes	Yes	Yes	Yes	Yes	9/13
Gholami et al. (2024) [52]	Yes	Yes	Yes	No	No	Yes	Yes	Yes	Yes	Yes	Yes	Yes	Yes	11/13
Nouryan et al. (2019) [56]	Yes	N/A	Yes	No	No	N/A	Yes	Yes	Yes	Yes	Yes	Yes	Yes	9/13

N/A = not available.

**Table 3:** Summary of the critical appraisal of the methodology of the quasi-experimental studies included in the review (*n* = 2).

	Difference Between “Cause” and “Effect”	There Is a Control Group	Compared Participants Were Similar	Similar Treatment/Care	Multiple Pre- and Post-Intervention Outcome Measures	Same Outcome Measurement in Both Groups	Reliable Measurements of Results	Was the Follow-Up Completed or Were the Differences Between Groups Adequately Described and Analysed?	Appropriate Statistical Analysis	Total Quality Score
Radhakrishnan et al. (2016) [47]	No	No	N/A	N/A	Yes	N/A	Yes	Yes	Yes	4/9
Donesky et al. (2017) [53]	No	Yes	Yes	No	Yes	Yes	Yes	Yes	Yes	7/9

N/A = not available.

**Table 4:** Summary of the critical appraisal of the methodology of the qualitative studies included in the review (*n* = 1).

	Is There Congruity Between the Philosophical or Theoretical Positioning of the Study and the Methodological Approach That Has Been Adopted?	Is There Congruity Between the Research Methodology and the Research Question or Objectives?	Is There Congruity Between the Research Methodology and the Methods Used to Collect Data?	Is There Congruity Between the Research Methodology and the Representation and Analysis of Data?	Is There Congruity Between the Research Methodology and the Interpretation of Results?	Is There a Statement Locating the Researcher Culturally or Theoretically?	Is the Influence of the Researcher on the Research, and Vice Versa, Addressed?	Are Participants, and Where Appropriate, Their Voices Adequately Represented?	Is the Research Ethical According to Current Criteria, and for Recent Studies, Is There Evidence of Ethical Approval by an Appropriate Body?	Do the Conclusions Drawn in the Research Report Flow from the Analysis, or Interpretation, of the Data?	Total Quality Score
Yeh et al. (2016) [50]	Yes	Yes	Yes	Yes	Yes	N/A	N/A	Yes	Yes	Yes	8/10

N/A = not available.

**Table 5:** Summary of the critical appraisal of the methodology of the cohort studies included in the review (*n* = 4).

	Were the Two Groups Similar and Recruited from the Same Population?	Were the Exposures Measured Similarly to Assign People to Both Exposed and Unexposed Groups?	Was the Exposure Measured in a Valid and Reliable Way?	Were Confounding Factors Identified?	Were Strategies to Deal with Confounding Factors Stated?	Were the Groups/Participants Free of the Outcome at the Start of the Study (or at the Moment of Exposure)?	Were the Outcomes Measured in a Valid and Reliable Way?	Was the Follow-Up Time Reported and Sufficient to Be Long Enough for Outcomes to Occur?	Was Follow-Up Completed, and If Not, Were the Reasons for Loss to Follow-Up Described and Explored?	Were Strategies to Address Incomplete Follow-Up Utilized?	Was Appropriate Statistical Analysis Used?	Total Quality Score
Lind et al. (2016) [54]	No	N/A	Yes	N/A	No	Yes	Yes	Yes	Yes	N/A	N/A	5/11
Gokalp et al. (2018) [55]	No	No	Yes	N/A	No	Yes	Yes	Yes	N/A	No	N/A	8/11
Persson et al. (2020) [57]	N/A	N/A	Yes	Yes	Yes	Yes	Yes	Yes	Yes	N/A	Yes	8/11

N/A = not available.

## 3. Results

### 3.1. Characteristics and Samples of the Study

The articles included in this review (*n* = 12) involved a total of 2466 community-dwelling older adults with CHF. Patients belonging to all four functional classes established by the New York Heart Association (NYHA) were included. These articles were published between 2014 and 2024.

Among the studies, there were randomised controlled trials (*n* = 6), cohort studies (*n* = 3), quasi-experimental studies (*n* = 2), and qualitative studies (*n* = 1).

### 3.2. Domains of Intervention

Although all the studies included in this review attempt to address different factors affecting older adults with CHF, the interventions can be grouped into 4 domains: education/cognitive (EDU), physical activity (PA), telehealth (TH), and nutrition (N).

### 3.3. Programmes Implemented and Evaluated

Several interventions were designed to improve biopsychosocial aspects of community-dwelling older adults with CHF. These interventions were executed under the supervision of nurses (*n* = 14), physicians/cardiologists (*n* = 2), physiotherapists (*n* = 3), health educators (*n* = 1), specialised sports instructors (*n* = 2), and occupational therapists (*n* = 1), constituting multidisciplinary teams in most cases. Below, we explain how each intervention was carried out, grouping the studies based on the established domains.

#### 3.3.1. EDU-Self-Care

Dickson et al. [46] implemented a bi-weekly educational intervention to generate knowledge, quality of life, and self-care capabilities. For 4 weeks, an intervention group (IG) attended the intervention, and a control group (CG) was assigned to usual care.

#### 3.3.2. EDU-Video Game

Radhakrishnan et al. [47] developed a digital game prototype aiming to increase the patient’s knowledge of CHF alongside their self-care capacity. The subjects provided feedback on the use of the system for its future refinement.

#### 3.3.3. EDU-Telematics

Kalter-Leibovici [48] aimed to determine the effectiveness of a multidisciplinary disease management programme after implementing it for 6 months. A CG was assigned to usual visits to their cardiologist. The IG maintained remote contact via telephone and/or video call with their PC professional, alongside clinic visits during the process.

#### 3.3.4. PA-Multimodal Exercise

Antonicelli et al. [49] established an exercise programme consisting of 3 weekly sessions for 3 months, initially in person and subsequently via telematics from home. The Training Care Group (TCG) was assigned to the protocol, and the remaining subjects were placed in the usual care group (UCG) until the end of the programme at 12 months.

#### 3.3.5. PA-Tai Chi

Yeh et al. [50] conducted guided Tai Chi sessions to explore their physical and psychological effects. An education group received bi-weekly basic group education sessions, and the Tai Chi group performed bi-weekly sessions alongside individual home sessions 3 times a week. At 12 weeks, interviews were conducted to gather information regarding the experience.

#### 3.3.6. PA-Reach

Dibben et al. [51] aimed to evaluate the impact of home-based cardiac rehabilitation on the physical activity of patients with heart failure and to explore the factors influencing activity changes. Part of the sample was assigned to a CG and received usual care. The REACH-HF group received a self-management programme with a manual, and exercise was recommended 3 or more times per week.

#### 3.3.7. PA-Balance

Gholami et al. [52] aimed to determine the effect of supervised balance training on cognitive function and activities of daily living (ADLs). One group was assigned to usual care (UC) with sporadic visits to their cardiologist. The balance training (BT) group performed 4 weekly sessions of 30 min during the 8-week intervention.

#### 3.3.8. PA + TH-Tele-Yoga

Donesky et al. [53] allocated 14 patients into two groups for a tele-yoga intervention. The control group was provided with educational material and received weekly nursing telephone calls. The tele-yoga group attended 2 online classes per week for the 8-week duration of the project.

#### 3.3.9. TH-Digital Pen

Lind et al. [54] implemented a telemonitoring and vital signs recording system for one year. These data were reviewed and processed by nurses. The nurses conducted home visits and referred patients to other specialists based on the information obtained.

#### 3.3.10. TH-Integrated Monitoring

Gokalp et al. [55] investigated the potential of an integrated care system that acquires data on clinical signs and vital habits to support independent living in older people with chronic conditions. Telemonitoring systems were set up in the patients’ homes alongside thresholds that alerted their PC nurses in cases of abnormal patterns, enabling timely intervention. The programme managed to extend up to 495 days of use for one of the subjects.

#### 3.3.11. TH-Monitoring + Consultations

Nouryan et al. [56] aimed to compare emergency department visits, hospitalisations, length of stay, quality of life, and costs between two groups over a 6-month period. One group (COM) was assigned to usual care. The intervention group (HTM) conducted weekly tele-consultations and maintained daily vital signs monitoring. At the end of the programme, the differences between both groups were recorded.

#### 3.3.12. TH-Health Diary

Persson et al. [57] evaluated whether the Health Diary telemonitoring system combined with hospital home care improves health-related quality of life in older people with CHF. The patients committed to a daily log of symptoms and vital signs until the end of the study, alongside on-demand contact with PC. Finally, the reduction in the use of healthcare services and hospitalisations was calculated.

### 3.4. Characteristics (Duration and Frequency) of the Programmes

The reviewed programmes varied in duration, ranging from 4 weeks to 16 months. Most programmes were implemented for no longer than a year, reaching up to 495 days in the case of TH-Integrated monitoring [55]. The EDU-Self-care [46], TH-Digital pen [54], and EDU-Video game [47] interventions lasted 4 weeks. The PA + TH-Tele-yoga [53] and PA-Balance [52] programmes took place over 8 weeks. The PA-Tai Chi [50] intervention was conducted over 12 weeks. The PA-Multimodal Exercise [49], EDU-Telematics [47], and TH-Monitoring + consultations [56] interventions were carried out over 6 months. Finally, the PA-Reach [51] and TH-Health Diary [57] programmes had a duration of 12 months.

Most studies collected data up to the end of the intervention period, with the exception of four that conducted a subsequent follow-up. The EDU-Self-care [46] and TH-Digital pen [54] programmes (4 weeks) carried out follow-ups at 3 and 12 months from project inception, respectively. EDU-Telematics [48] (6 months) performed several measurements at 12, 18, and 24 months from the start of the intervention. Furthermore, TH-Integrated monitoring [55], TH-Monitoring + consultations [56], and TH-Health Diary [57] measured and evaluated patient vital signs and symptomatology on a daily basis.

### 3.5. Evaluated Results

#### 3.5.1. Physical-Functional

Several authors (*n* = 3) [48,49,53] applied the 6 min walk test (6MWT) as a method to measure physical function and/or endurance. After 8 weeks of intervention, PA + TH-Tele-yoga [53] detected that members of the yoga intervention reported less dyspnoea and breathing difficulty during walking compared to those in the CG (*p* = 0.03). PA-Multimodal Exercise [49] reported better results in the TCG at 3 months compared to the UCG (*p* = 0.001). There were no differences between the two groups in EDU-Telematics [48]. Patient strength was assessed by PA + TH-Tele-yoga [53]; the research team quantified how many bicep curls or sit-to-stands a subject could perform in 30 s and found no improvements between the baseline and the end of the programme. The PA-Multimodal Exercise [49] measured levels of NT-proBNP, a prohormone released in response to cardiac overload. It was observed that in the TCG, levels decreased considerably at 6 months. PA-Reach [51] measured physical activity levels using the GeneActiv accelerometer. The results showed that within a 12-month timeframe, the REACH-HF group increased light activity time and decreased sedentary time compared to the CG. Patients under the Tai Chi intervention in PA-Tai Chi had increased their level of moderate physical activity by the time the programme concluded. The levels of dyspnoea experienced by patients were measured in PA + TH-Tele-yoga [53]. No differences were found between the groups at the end of the intervention. Functional capacity to perform activities of daily living (ADLs) and instrumental activities of daily living (IADLs) were evaluated in two studies [49,52]. PA-Multimodal Exercise [49] established a greater increase in ADLs in the CG than the IC at 6 months. Conversely, PA-Balance [52] indicated a greater improvement in both functions in the BT group compared to the CG within just 8 weeks from the start of the intervention.

#### 3.5.2. Psychological

Patient depression levels were measured in two studies [48,53]. PA + TH-Tele-yoga [53] used the Patient Health Questionnaire-8 (PHQ-8), while EDU-Telematics [48] utilised the PHQ-9. Both studies observed that the IGs showed a decrease in depressive symptoms, whereas these symptoms worsened in the CGs. PA + TH-Tele-yoga [53] evaluated insomnia using the General Sleep Disturbance Scale. This decreased significantly in the IG compared to the CG at the end of the intervention, following the 8-week programme. Conversely, PA-Balance [52] employed the Montreal Cognitive Assessment (MoCA-B) and indicated that upon completion of the intervention, the BT group exhibited significantly higher cognitive function than the group assigned to usual care (*p* = 0.001). Individuals undergoing the Tai Chi intervention in PA-Tai Chi [50] reported greater overall relaxation and calmness cultivated through the exercise, which helped them alleviate stress. They also described a newfound capacity to act with greater resilience, displaying less automaticity when generating emotional responses to difficult situations or stressors. Alongside this, they reported an increased or novel sense of mindfulness. Participants described an awareness of their breathing, as well as of their own bodily sensations, cues, and symptoms, and how this enabled them to forge a new connection with themselves.

#### 3.5.3. Quality of Life (QoL)

QoL was measured in six of the articles using up to four different tools [46,48,49,53,56,57]. QoL was assessed both generically and specifically in relation to heart disease. The Kansas City Cardiomyopathy Questionnaire (KCCQ) was employed by EDU-Self-care [46] and PA + TH-Tele-yoga [53]. There were no significant differences between the IG and CG in the post-intervention measurements of either EDU-Self-care or PA + TH-Tele-yoga, with both groups showing a slight increase in both cases.

The Minnesota Living with Heart Failure Questionnaire (MLHFQ) yielded findings across several studies. In the PA-Multimodal Exercise intervention [49], both groups (TCG and UCG) showed an increase in their QoL at 6 months, which was substantially greater in the TCG. A similar pattern was observed in TH-Monitoring + consultations [56], where QoL increased in both groups but was much higher in the HTM group at the 6-month measurement. TH-Health Diary [57] indicated an improvement in disease-specific QoL among subjects undergoing telemonitoring.

Generic QoL was measured using the Short Form 36-item health survey (SF-36) in two studies [48,57]. According to EDU-Telematics [47], patients in the IG were more likely to experience improvements in quality of life than those assigned to usual care.

TH-Health Diary [57] combined the SF-36 with the EQ-5D, with patients in the intervention group demonstrating notable improvements in physical role functioning, social functioning, and mental health.

#### 3.5.4. Self-Care and Health-Literacy

Self-care capacity was addressed in two of the studies [46,47]. EDU-Self-care [46], using the Self-Care of Heart Failure Index (SCHFI), established an increase in the IG compared to the CG when measured one month after the start of the intervention (*p* = 0.04). This difference became more pronounced at the 3-month follow-up, showing a further increase in capacity within the IG and a plateau in the CG. Conversely, EDU-Video game [47] found no significant difference when applying the SCHFI to compare subjects’ measurements at baseline and at 4 weeks. The level of knowledge regarding CHF was measured in two studies [46,47]. EDU-Self-care [46] established a higher level of knowledge in the IG using the Dutch HF Knowledge Scale (DHFKS) at 3 months from the start of the intervention (*p* = 0.001). EDU-Video game [47] indicated an increase in knowledge 4 weeks after commencing the intervention (*p* = 0.007). The studies most reliant on new technologies (*n* = 3) measured adherence to system use (accesses or vital signs logged per day) [47,54,55]. The TH-Digital pen study [54] successfully achieved daily logging of vital signs and symptoms by all patients, whereas only 60% of patients in TH-Integrated monitoring [55] recorded their blood pressure daily. These programmes based on the use of ICTs in healthcare also measured the interest or utility perceived by the patient. The older adults who trialled the TH-Digital pen system [54] demonstrated on Likert scales that the system was simple to use. Similarly, the subjects in EDU-Video game [47] found the system interesting, enjoyable, and easy to use, as reported in the subscale of the Intrinsic Motivation Inventory employed.

#### 3.5.5. Healthcare Utilisation and Costs

Five studies measured patient hospital admissions during their execution [48,49,54,56,57]. PA-Multimodal Exercise [49] recorded that the TCG had less than half the proportion of hospital admissions compared with the group assigned to basic care (*p* = 0.001). None of the patients in TH-Digital pen [54] experienced readmissions. EDU-Telematics [48] detected that patients in the IG took, on average, approximately one year longer to be readmitted than those in the CG. TH-Monitoring + consultations [56] indicated no differences between both groups. Finally, TH-Health Diary [57] compared the number of hospitalisations during the 12-month intervention with those in the previous year, reporting a 74% reduction. The use of emergency services was recorded by two investigators [56,57]. TH-Monitoring + consultations [56] detected more emergency department visits in COM vs. HTM: 60% vs. 38% (*p* = 0.004). TH-Health Diary [57] detected an average of 0.8 visits per patient. Finally, two studies measured the estimated per capita expenditure of the study subjects [56,57]. TH-Health Diary [57] estimated a mean expenditure of €21,515 during the year the project lasted (*p* = 0.01). TH-Monitoring + consultations [56] took a different approach and established that the cost of implementing the intervention for one year was $1600, significantly lower in comparison with the average of $38,990 for usual care. The monitoring in TH-Integrated monitoring [55] enabled the premature detection of acute exacerbations. This led to an increase in diagnoses and specialised interventions, thereby achieving early care and preventing complications that would not be detectable with conventional care.

### 3.6. Results of the Critical Appraisal

The methodological quality of the included studies was assessed using the Joanna Briggs Institute (JBI) critical appraisal tools, according to study design [43,44,45]: randomised controlled trials (RCTs), quasi-experimental studies, cohorts, and qualitative studies.

The critical appraisal tool for RCTs [42] was used in 6 of the included studies [46,48,49,51,52,56]. The mean scores of the RCTs ranged from 9 to 11 out of a total of 13 items, indicating fair-to-good quality. All studies carried out true randomisation, along with the correct use of outcome variables and appropriate statistical analysis. However, most did not apply blinding to administrators and participants. One of these studies did not specify whether an evaluation of subjects lost to follow-up during the intervention was conducted (intention-to-treat analysis), which affects the reliability of the results [49] (Table 2).

The critical appraisal tool for quasi-experimental studies [44] was used in 2 of the included studies [47,53]. Their scores were 4 and 7 points out of 9 items, indicating poor-to-good quality. Both established multiple pre- and post-intervention outcome variables, reliable outcome measures, and complete follow-ups. Radhakrishnan [47], being a feasibility study, lacked a control group. Donesky et al. [53] did not establish a correct distinction between cause and effect (Table 3).

The critical appraisal tool for qualitative studies [45] was used in 1 of the included studies [50]. This study scored 8 out of 10 items, indicating good quality. Congruence was established between the methodology and the analysis and representation of information; the participants were adequately represented, and evidence of ethical committee approval was shown. There was no statement locating the researcher culturally or theoretically, nor was the researcher’s influence on the study addressed (Table 4).

The critical appraisal tool for cohort studies was used in 3 of the included studies [54,55,57]. The mean scores ranged from 5 to 8 out of a total of 11 items, indicating poor-to-good quality. In all of them, exposure was measured in a valid and reliable manner. In two of them, it was not specified whether an appropriate statistical analysis was used [54,55]. None of the studies utilised strategies to address incomplete follow-up, nor did they use two similar groups recruited from the same population (Table 5).

## 4. Discussion

The first specific objective of this review was to identify the various non-pharmacological interventions available for community-dwelling older adults with CHF. Our findings in this regard demonstrate that the implemented programmes exhibit considerable variability. Several programmes focused on physical activity, encompassing everything from balance exercises to yoga, while including strength and aerobic endurance training. These findings align with the substantial body of current evidence associating sports practice with an improvement in physico-functional variables [58,59,60]. The evidence proposed by other authors when implementing this type of intervention suggests that patients not only improve physical parameters but also experience enhancements in their quality of life and psychological well-being [61]. Other studies evaluated patient education-based interventions, as seen in EDU-Self-care [45] and EDU-Video game [47]. This is consistent with previous research that advocated for the importance of enhancing self-care capacity and disease knowledge [62].

Several of the included studies implemented interventions based on the use of telecare and new technologies (TH-Digital pen; TH-Integrated monitoring) [54,55]. In recent years, there has been an upsurge in research within this field, coinciding with improved access to and use of these technologies by the general public [63,64,65]. These studies could be particularly relevant in the current context, thus reinforcing the need to adapt digital media to existing practices and systems in the care of older adults with CHF [66]. These findings are highly relevant to nursing practice, not only because they highlight the sheer volume of current research addressing this topic, but also because they showcase the different avenues of action available for treating older adults with CHF.

The second specific objective of this review was to describe the main characteristics, duration, and frequency of the programmes. Following an evaluation of the results, it was observed that programme duration influenced the improvement of several factors, which is consistent with current evidence [67]. Those programmes more closely focused on physical activity seemed to show better results when the interventions were extended over longer periods. This aligns with the evidence put forward by other studies [68]. Several authors hypothesise that this may be due to the limited metabolic reserve of patients with advanced-stage CHF, who require more protracted intervention processes to achieve positive outcomes [69,70].

On the other hand, the programmes that measured psychological factors after targeting them directly seem to have obtained positive results regardless of programme duration, which is consistent with recent publications [71,72]. Several authors suggest that this may occur because a decline in leisure and/or intellectual activities in older adults gives rise to depressive symptomatology and cognitive impairment. These interventions provide patients with a sense of purpose or a pastime that mitigates this issue [73,74]. Those studies that measured improvements in self-care capabilities and knowledge seem to show that the primary influential factor was the number of sessions and repeated contact by healthcare personnel. These findings coincide with current evidence, highlighting the importance of repeated check-ups and continuity of care to achieve increased adherence to established treatments [75,76]. All these results are particularly relevant to nursing practice because they provide insight into the intervention timeframes required depending on the factors targeted for improvement. This allows for the simultaneous implementation of multiple interventions targeting different domains, rather than the execution of large-scale programmes that lack depth in certain areas. This approach, combined with periodic reviews to boost adherence, could significantly enhance both effectiveness and efficiency when treating patients.

The third specific objective of this review was to evaluate the measured outcomes in the selected studies. The primary results were grouped into several categories, including physico-functional factors, psychological factors, quality of life, health literacy, and healthcare utilisation/costs. Regarding physico-functional factors, our review found variations between studies that may stem from the nature of the interventions. For instance, studies focused on patient education might increase their level of knowledge about the disease, but they do not appear to directly improve factors such as endurance and balance in the way that physical activity-focused programmes do (EDU-Self-care) [46]. This aligns with recent evidence indicating a need to implement specific interventions tailored to the variables under treatment [77].

In some of these physical activity studies, greater improvements were observed relative to others; this could be attributed to sample variability between studies. The main influential factor might be associated with the differing NYHA functional classes of the participating patients. While some studies work with samples predominantly in classes II and III (PA-Reach) [51], others feature subjects with advanced class III and class IV heart failure. Numerous authors point out the difficulty of improving physical markers in advanced stages of CHF belonging to NYHA classes III and IV [70]. This difficulty is explained by several authors as a lack of physiological reserves sufficient to bring about a notable change [69].

The psychological domain improved in the majority of the studies that evaluated it, reporting reductions in factors such as depression and insomnia (PA + TH-Tele-yoga; EDU-Telematics) [48,53]. Other programmes showed improvements in general calmness and relaxation levels (PA-Tai Chi) [50]. These findings concur with recent authors who advocate for these interventions due to the psychological improvements experienced in both the short and long term [61,72].

With respect to quality of life, our review suggests that these types of programmes may improve quality of life, as reported in several studies across the PA, EDU, and TH domains. A few papers did not report improvements in this aspect, as was the case for EDU-Self-care [46] and PA + TH-Tele-yoga [53]. This situation is reflected in current evidence, with some authors defending the capacity of these interventions to increase QoL whereas others have found no significant improvements [74,78]. Some researchers suggest that this discrepancy may be due to the scales employed. Certain scales measure predominantly physical categories while others evaluate more psychological factors, suggesting that the choice of instrument can skew the perception of QoL [79].

Our review suggests that health literacy improved following the implementation of these programmes. An increase in self-care capacity and condition-specific knowledge was indicated following the EDU-Self-care and EDU-Video game interventions [46,47]. Current evidence aligns with these observations, pointing to the potential value of educating patients so they can manage their own health status better [73]. Consequently, this approach may contribute to a reduction in the number of long-term complications and hospitalisations [80], representing a promising possibility that warrants further robust validation.

In terms of healthcare utilisation and costs, it is not possible to extract definitive figures that guarantee the cost-effectiveness of these interventions, given that this was only measured in two of the studies in this review (TH-Health Diary; TH-Monitoring + consultations) [56,57]. Both studies suggested a potential reduction in expenditure and/or increased efficiency compared to traditional care. Other included studies pointed towards a possible decrease in the number of hospital readmissions (PA-Multimodal Exercise; EDU-Telematics; TH-Digital pen; TH-Health Diary) [48,49,54,57]. These findings are present in the current literature, albeit with limited backing due to sparse research and a lack of standardisation [81]. Nonetheless, some studies highlight the cost-effectiveness of these programmes, suggesting lower expenditure might be linked to reduced use of healthcare services [82,83]. Several authors support the feasibility and economic performance of these interventions, providing a rationale for their widespread implementation [80].

Owing to all of the above, nurses should exercise careful consideration when selecting lines of action and designing care plans for community-dwelling older adults with CHF. This information could assist them in targeting potential biopsychosocial improvements, while also reducing expenditure and the financial burden placed on healthcare systems. The findings of this integrative review suggest the potential viability of health programmes based on non-pharmacological nursing interventions to confront the challenges faced by community-dwelling older adults with CHF. These programmes could be structured to address multiple factors affecting the lives of these individuals, ranging from physical to psychological, social, and economic domains [84,85,86]. In turn, these issues can be approached from different perspectives, utilizing programmes based on activity, education, and follow-up monitoring, among others [62,87,88].

While this integrative review synthesises valuable pathways for non-pharmacological nursing care, the substantial methodological heterogeneity across the remaining included literature inherently limits the direct comparability of findings and the overall generalisability of our conclusions. This variance is most visibly pronounced in the sample sizes, which span from smaller-scale cohorts to a large sample of 1360 individuals in the multidisciplinary trial by Kalter-Leibovici et al. [48]. This wide range introduces critical discrepancies in statistical power and effect size interpretation. Smaller studies risk being underpowered, making them more susceptible to Type II errors or overestimating effect sizes due to sampling variability. In contrast, highly powered, large-scale trials firmly establish statistical significance, yet their broad, aggregate data may mask nuance regarding which specific patient profiles benefit most from targeted nursing care.

Beyond sample sizes, clinical and operational differences further confound comparisons across the literature. The inclusion of varying severity levels across the New York Heart Association (NYHA) functional classes complicates synthesis; interventions that prove highly successful for more stable patients (NYHA classes II–III) in certain protocols may show altered or restricted efficacy when applied to advanced, low-reserve cohorts (NYHA classes III–IV) featured in other studies. Similarly, the use of up to four distinct metrics to quantify Quality of Life (such as the disease-specific MLHFQ and KCCQ versus the generic SF-36 and EQ-5D) means that the identified benefits are viewed through different evaluative lenses, clouding direct comparisons of patient-reported outcomes. Finally, the diverse intervention windows and follow-up durations—ranging from a brief 4 weeks [46,47] to active periods over a year [51,55,57]—leave it unclear whether the documented biopsychosocial improvements can be sustained as permanent behavioral changes or if they are short-term responses that fade shortly after nursing contact concludes. Consequently, these layered variations necessitate a cautious interpretation of the collective data and highlight the need for standardized clinical protocols in future research.

### Limitations and Future Research

This integrative review has several limitations. We excluded studies on interventions that, despite being non-pharmacological and targeting older adults aged over 60 with CHF, were not conducted entirely within the community setting. Some of these studies commenced briefly within the hospital setting, utilising hospital readmissions for recruitment and to carry out the initial phases of the intervention. It is highly probable that some of these programmes were methodologically similar to those selected, and it would be of interest to analyse their outcomes.

Similarly, we excluded studies of non-pharmacological interventions in community-dwelling older adults with cardiovascular disease who did not present specific outcome data for CHF. Some of the interventions in those programmes might well be applicable to our study population, making the analysis of their results a worthwhile pursuit. Furthermore, several of the selected papers exhibit limited methodological quality, which may cast some doubt on the robustness of their reported findings. In particular, some studies lacked comparable control groups, did not adequately address incomplete follow-up, or provided limited information regarding statistical analyses. These methodological shortcomings may affect the reliability of the evidence synthesised and introduce potential bias into the overall conclusions of the review. The inclusion of additional databases such as EMBASE could have enriched the search. Inter-rater agreement was not formally quantified during screening. Prospective calculation of Cohen’s kappa is recommended for future reviews.

Given the diverse approaches to intervening with these patients, future research should focus on designing action plans that enable the development of targeted interventions to address the various limiting factors present in this population. At the same time, it is necessary to explore the long-term effects of these interventions and to identify the mechanisms that enhance their effectiveness. Understanding which elements contribute to favourable outcomes will be pivotal for optimising existing programmes and creating novel proposals tailored to the needs of community-dwelling older adults with CHF [89]. Finally, it is essential to analyse the cost-effectiveness of these programmes compared to usual care, with the aim of achieving greater efficiency in clinical practice.

## 5. Conclusions

The objective of this integrative review was to synthesise and integrate the implemented and evaluated nursing interventions aimed at improving the biopsychosocial factors of community-dwelling older adults with Chronic Heart Failure. The results suggest a noticeable diversity in both the reported outcomes and the design of the interventions. Educational and telecare-based interventions appeared to show promising effects, particularly on psychological and cognitive outcomes, whereas interventions focused on physical activity yielded less consistent results. However, the cost-effectiveness and efficiency of these programmes compared with usual care remain insufficiently established and require confirmation through high-certainty evidence.

Despite the heterogeneity of the programmes and the variability in the methodological quality of the studies, the findings suggest the potential viability of exploring tailored interventions to improve the health of community-dwelling older adults with CHF. Nevertheless, the long-term effectiveness, sustainability, and economic value of these interventions remain uncertain. Future well-designed, standardised studies incorporating robust economic evaluations are needed to determine their clinical effectiveness, cost-effectiveness, and potential for implementation in routine practice.

## Data Availability

No new data were created or analyzed in this study.

## Appendix A

**Table A1:** Keywords and natural language.

Keyword	Medical Subject Headings (MeSH)	Natural Language (Translation)
Elderly	Aged	Adult Over 65 years of age
Older adults		
Aged		
Older		
Cardiac Failure	Congestive Heart Failure	Congestive Heart Failure
Heart Failure, Congestive		
Myocardial Failure		
Living, Independent	Community Dwelling	Community Dwelling
Aging in Place		
Dwelling, Community		
Home Environment *		
Living at home		
Intervention *		Intervention/Program
Program *		
Programme *		
Non-pharmacological		
Physical Exercise	Exercise	Exercise
Physical Activity		
Exercises		
Patient Cooperation	Patient Compliance	Adherence
Patient Adherence		
Treatment Compliance		
Therapeutic Compliances		
Client Adherence		
Client Compliance		
Behaviors		
Behavioral		
Cognitive Behavioral Therapy		
Behavioral Therapy, Cognitive		
Cognitive Behavioral Therapies	Behavioral	Behavioral
Therapies, Cognitive Behavior		
Cognition Therapy		
Cognitive Therapy		
Cognitiv *		
Therapy, Cognitive		
Nutr *	Nutrition Policy	Nutrition
Nutrition Policies		
Food Policy		
Dietary Guideline *		
Guideline, Dietary		
Nutrition, Guideline *		
Nutraceutical		
Rehabilitation	Rehabilitation	Rehabilitation
Rehab*		

(*) indicates truncation and is used to retrieve words with different endings.

**Table A2:** Search strategy and results for each database.

Database	Search Strategy (Title-Abs-Key)	Results
PubMed	((aged) OR (Elderly) OR (Older adults) OR (Older)) AND ((Congestive Heart Failure) OR (Cardiac Failure) OR (Heart Failure, Congestive) OR (Myocardial Failure)) AND ((Living, Independent) OR (Aging in Place) OR (Community Dwelling) OR (Dwelling, Community) OR (Dwellings, Community) OR (Home Environment*) OR (Living at home)) AND ((Patient compliance) OR (Patient Cooperation) OR (Patient Adherence) OR (Treatment Compliance) OR (Therapeutic Compliances) OR (Client Adherence) OR (Client Compliance) OR (Exercise) OR (Physical Exercise) OR (Physical Activity) OR (Exercise Training) OR (Exercises) OR (Behavioral) OR (Behaviors) OR (Cognitive Behavioral Therapy) OR (Behavioral Therapy, Cognitive) OR (Cognitive Behavioral Therapies) OR (Therapies, Cognitive Behavior) OR (Cognition Therapy) OR (Cognitive Therapy) OR (Cognitiv*) OR (Therapy, Cognitive) OR (nutrition) OR (Nutr*) OR (Nutrition Policies) OR (Food Policy) OR (Dietary Guideline*) OR (Guideline, Dietary) OR (Nutrition Guideline*) OR (Nutraceutical) OR (Non-pharmacological) OR (Rehabilitation) OR (Rehab*)) AND ((Intervention*) OR (Program*) OR (Programme*))	326
Cochrane	(aged OR elderly OR “older adults” OR older) AND (“congestive heart failure” OR “cardiac failure” OR “heart failure, congestive” OR “myocardial failure”) AND (“living, independent” OR “aging in place” OR “community dwelling” OR “dwelling, community” OR “dwellings, community” OR “home environment*” OR “living at home”) AND (“patient compliance” OR “patient cooperation” OR “patient adherence” OR “treatment compliance” OR “therapeutic compliances” OR “client adherence” OR “client compliance” OR exercise OR “physical exercise” OR “physical activity” OR “exercise training” OR exercises OR behavioral OR behaviors OR “cognitive behavioral therapy” OR “behavioral therapy, cognitive” OR “cognitive behavioral therapies” OR “therapies, cognitive behavior” OR “cognition therapy” OR “cognitive therapy” OR cognitiv* OR “therapy, cognitive” OR nutrition OR nutr* OR “nutrition policies” OR “food policy” OR “dietary guideline*” OR “guideline, dietary” OR “nutrition guideline*” OR nutraceutical OR “non-pharmacological” OR rehabilitation OR rehab*) AND (intervention* OR program* OR programme*)	6
CINHAL	(aged OR elderly OR “older adults” OR older) AND (“congestive heart failure” OR “cardiac failure” OR “heart failure, congestive” OR “myocardial failure”) AND (“living, independent” OR “aging in place” OR “community dwelling” OR “dwelling, community” OR “dwellings, community” OR “home environment*” OR “living at home”) AND (“patient compliance” OR “patient cooperation” OR “patient adherence” OR “treatment compliance” OR “therapeutic compliances” OR “client adherence” OR “client compliance” OR exercise OR “physical exercise” OR “physical activity” OR “exercise training” OR exercises OR behavioral OR behaviors OR “cognitive behavioral therapy” OR “behavioral therapy, cognitive” OR “cognitive behavioral therapies” OR “therapies, cognitive behavior” OR “cognition therapy” OR “cognitive therapy” OR cognitiv* OR “therapy, cognitive” OR nutrition OR nutr* OR “nutrition policies” OR “food policy” OR “dietary guideline*” OR “guideline, dietary” OR “nutrition guideline*” OR nutraceutical OR “non-pharmacological” OR rehabilitation OR rehab*) AND (intervention* OR program* OR programme*)	66
Web of Science	TS = (“aged” OR “elderly” OR “older adults” OR “older”) AND TS = (“congestive heart failure” OR “cardiac failure” OR “heart failure, congestive” OR “myocardial failure”) AND TS = (“living, independent” OR “aging in place” OR “community dwelling” OR “dwelling, community” OR “dwellings, community” OR “home environment*” OR “living at home”) AND TS = (“patient compliance” OR “patient cooperation” OR “patient adherence” OR “treatment compliance” OR “therapeutic compliances” OR “client adherence” OR “client compliance” OR “exercise” OR “physical exercise” OR “physical activity” OR “exercise training” OR “exercises” OR “behavioral” OR “behaviors” OR “cognitive behavioral therapy” OR “behavioral therapy, cognitive” OR “cognitive behavioral therapies” OR “therapies, cognitive behavior” OR “cognition therapy” OR “cognitive therapy” OR “cognitiv*” OR “therapy, cognitive” OR “nutrition” OR “nutr*” OR “nutrition policies” OR “food policy” OR “dietary guideline*” OR “guideline, dietary” OR “nutrition guideline*” OR “nutraceutical” OR “non-pharmacological” OR “rehabilitation” OR “rehab*”) AND TS = (“intervention*” OR “program*” OR “programme*”)	8
SCOPUS	(aged OR elderly OR “older adults” OR older) AND (“congestive heart failure” OR “cardiac failure” OR “heart failure, congestive” OR “myocardial failure”) AND TITLE-ABS-KEY(“living, independent” OR “aging in place” OR “community dwelling” OR “dwelling, community” OR “dwellings, community” OR “home environment *” OR “living at home”) AND (“patient compliance” OR “patient cooperation” OR “patient adherence” OR “treatment compliance” OR “therapeutic compliances” OR “client adherence” OR “client compliance” OR exercise OR “physical exercise” OR “physical activity” OR “exercise training” OR exercises OR behavioral OR behaviors OR “cognitive behavioral therapy” OR “behavioral therapy, cognitive” OR “cognitive behavioral therapies” OR “therapies, cognitive behavior” OR “cognition therapy” OR “cognitive therapy” OR cognitiv* OR “therapy, cognitive” OR nutrition OR nutr* OR “nutrition policies” OR “food policy” OR “dietary guideline*” OR “guideline, dietary” OR “nutrition guideline*” OR nutraceutical OR “non-pharmacological” OR rehabilitation OR rehab*) AND (intervention* OR program* OR programme*)	25
Google Scholar	intitle:“aged” OR intitle:“elderly” OR “older adults” OR “older” AND “congestive heart failure” OR “cardiac failure” OR “heart failure, congestive” AND intitle:“living, independent” OR “aging in place” OR “living at home” AND “patient compliance” OR “patient adherence” OR “treatment compliance” OR “client adherence” AND “exercise” OR “exercise training” OR “cognitive behavioral therapy” OR “therapy, cognitive” AND “nutrition” OR “nutr*” OR rehabilitation OR “rehab*” AND intitle:“intervention*” OR “program*” OR “programme*” OR “non-pharmacological”	240
	TOTAL:	671

(*) indicates truncation and is used to retrieve words with different endings.

## References

[1] Desiderio A., Pastorino M., Campitelli M., Longo M., Miele C., Napoli R., Beguinot F., Raciti G.A. (2024). DNA methylation in cardiovascular disease and heart failure: Novel prediction models?. Clin. Epigenet..

[2] Karaye K.M., Dokainish H., ElSayed A., Mondo C., Damasceno A., Sliwa K., Balasubramanian K., Grinvalds A., Yusuf S. (2021). Clinical Profiles and Outcomes of Heart Failure in Five African Countries: Results from INTER-CHF Study. Glob. Heart.

[3] Sicras-Mainar A. (2022). Epidemiología y tratamiento de la insuficiencia cardiaca en España: Estudio PATHWAYS-HF. Rev. Esp. Cardiol..

[4] Ponikowski P., Anker S.D., AlHabib K.F., Cowie M.R., Force T.L., Hu S., Jaarsma T., Krum H., Rastogi V., Rohde L.E. (2014). Heart failure: Preventing disease and death worldwide. ESC Heart Fail..

[5] McDonagh T.A. (2022). Guía ESC 2021 sobre el diagnóstico y tratamiento de la insuficiencia cardiaca aguda y crónica. Rev. Esp. Cardiol..

[6] Johansson I., Joseph P., Balasubramanian K., McMurray J.J.V., Lund L.H., Ezekowitz J.A., Kamath D., Alhabib K., Bayes-Genis A., Budaj A. (2021). Health-Related Quality of Life and Mortality in Heart Failure: The Global Congestive Heart Failure Study of 23 000 Patients from 40 Countries. Circulation.

[7] Niklasson A., Maher J., Patil R., Sillén H., Chen J., Gwaltney C., Rydén A. (2022). Living with heart failure: Patient experiences and implications for physical activity and daily living. ESC Heart Fail..

[8] Osenenko K.M., Kuti E., Deighton A.M., Pimple P., Szabo S.M. (2022). Burden of hospitalization for heart failure in the United States: A systematic literature review. J. Manag. Care Spec. Pharm..

[9] Scarà A., Palamà Z., Robles A.G., Dei L.-L., Borrelli A., Zanin F., Pignalosa L., Romano S., Sciarra L. (2024). Non-Pharmacological Treatment of Heart Failure—From Physical Activity to Electrical Therapies: A Literature Review. J. Cardiovasc. Dev. Dis..

[10] Wang Q.-C., Li J.-Y., Ni X.-S., Zhao W.-W., Wang X.-C., Wang L.-C. (2025). Impact of Walking and Respiratory Training on Cardiopulmonary Function and Activity Endurance in Patients with Chronic Heart Failure. Clin. Cardiol..

[11] Bozkurt B., Fonarow G.C., Goldberg L.R., Guglin M., Josephson R.A., Forman D.E., Lin G., Lindenfeld J., O’Connor C., Panjrath G. (2021). Cardiac Rehabilitation for Patients with Heart Failure: JACC Expert Panel. J. Am. Coll. Cardiol..

[12] Austin R.C., Schoonhoven L., Clancy M., Richardson A., Kalra P.R., May C.R. (2021). Do chronic heart failure symptoms interact with burden of treatment? Qualitative literature systematic review. BMJ Open.

[13] Celano C.M., Villegas A.C., Albanese A.M., Gaggin H.K., Huffman J.C. (2018). Depression and Anxiety in Heart Failure: A Review. Harv. Rev. Psychiatry.

[14] Harris K.M., Jacoby D.L., Lampert R., Soucier R.J., Burg M.M. (2021). Psychological Stress in Heart Failure: A Potentially Actionable Disease Modifier. Heart Fail. Rev..

[15] De Berardis D., Fornaro M., Orsolini L. (2020). Editorial: “No Words for Feelings, Yet!” Exploring Alexithymia, Disorder of Affect Regulation, and the “Mind-Body” Connection. Front. Psyquiatry.

[16] Olano-Lizarraga M., Wallström S., Martín-Martín J., Wolf A. (2022). Causes, experiences and consequences of the impact of chronic heart failure on the person’s social dimension: A scoping review. Health Soc. Care Community.

[17] Escobar C., Varela L., Palacios B., Capel M., Sicras A., Sicras A., Hormigo A., Alcázar R., Manito N., Botana M. (2020). Costs and healthcare utilisation of patients with heart failure in Spain. BMC Health Serv. Res..

[18] Heidenreich P.A., Bozkurt B., Aguilar D., Allen L.A., Byun J.J., Colvin M.M., Deswal A., Drazner M.H., Dunlay S.M., Evers L.R. (2022). 2022 AHA/ACC/HFSA Guideline for the Management of Heart Failure: A Report of the American College of Cardiology/American Heart Association Joint Committee on Clinical Practice Guidelines. Circulation.

[19] Bhatt A.S., Fonarow G.C., Greene S.J., Holmes D.N., Alhanti B., Devore A.D., Butler J., Heidenreich P.A., Huang J.C., Kittleson M.M. (2024). Medical Therapy Before, During and After Hospitalization in Medicare Beneficiaries with Heart Failure and Diabetes: Get with The Guidelines—Heart Failure Registry. J. Card. Fail..

[20] Felker G.M., Ellison D.H., Mullens W., Cox Z.L., Testani J.M. (2020). Diuretic Therapy for Patients with Heart Failure: JACC State-of-the-Art Review. J. Am. Coll. Cardiol..

[21] Xiang B., Yu Z., Zhou X. (2022). Comparative Efficacy of Medical Treatments for Chronic Heart Failure: A Network Meta-Analysis. Front. Cardiovasc. Med..

[22] Marti H.-P., Pavía López A.A., Schwartzmann P. (2024). Safety and tolerability of β-blockers: Importance of cardioselectivity. Curr. Med. Res. Opin..

[23] Xue Z., Liu X., Liu Q., Yang X., Yu L. (2025). A disproportionality analysis of adverse events associated with loop diuretics in the FDA Adverse Event Reporting System (FAERS). BMC Pharmacol. Toxicol..

[24] Anker S.D., Friede T., von Bardeleben R.-S., Butler J., Khan M.-S., Diek M., Heinrich J., Geyer M., Placzek M., Ferrari R. (2024). Transcatheter Valve Repair in Heart Failure with Moderate to Severe Mitral Regurgitation. N. Engl. J. Med..

[25] Zolotarova T.V., Brynza M.S., Volkov D.Y., Shevchuk M.I., Bilchenko O.V. (2021). Predictors of atrial fibrillation recurrence after radiofrequency ablation in patients with chronic heart failure. Wiad. Lek..

[26] Rivinius R., Heil K.M., Doesch A.O. (2023). Surgical ventricular reconstruction for the treatment of advanced heart failure—Return of the surgeons?. J. Thorac. Dis..

[27] Hirai T., Grantham J.A. (2021). Perforation Mechanisms, Risk Stratification, and Management in the Post-Coronary Artery Bypass Grafting Patient. Interv. Cardiol. Clin..

[28] McMenamin A., Turi E., Schlak A., Poghosyan L. (2023). A Systematic Review of Outcomes Related to Nurse Practitioner-Delivered Primary Care for Multiple Chronic Conditions. Med. Care Res. Rev..

[29] Bernard T.L., Hetland B., Schmaderer M., Zolty R., Pozehl B. (2023). Nurse-led heart failure educational interventions for patient and informal caregiver dyads: An integrative review. Heart Lung.

[30] Chen J., Jiang C., Guo M., Zeng Y., Jiang Z., Zhang D., Tu M., Tan X., Yan P., Xu X. (2024). Effects of SGLT2 inhibitors on cardiac function and health status in chronic heart failure: A systematic review and meta-analysis. Cardiovasc. Diabetol..

[31] Das B.B., Niu J. (2025). A Systematic Review and Meta-Analysis of the Safety and Efficacy of SGLT2 Inhibitors in Chronic Heart Failure in ACHD Patients. Am. J. Cardiovasc. Drugs.

[32] Kang H., Zhang J., Zhang X., Qin G., Wang K., Deng Z., Fang Y., Chen G. (2020). Effects of sacubitril/valsartan in patients with heart failure and chronic kidney disease: A meta-analysis. Eur. J. Pharmacol..

[33] Cochran J.M., Alam A., Guerrero-Miranda C.Y. (2022). Importance of right heart catheterization in advanced heart failure management. Rev. Cardiovasc. Med..

[34] Krzelj K., Petricevic M., Gasparovic H., Biocina B., McGiffin D. (2022). Ventricular Assist Device Driveline Infections: A Systematic Review. Thorac. Cardiovasc. Surg..

[35] Borghi-Silva A., Garcia-Araújo A.S., Winkermann E., Caruso F.R., Bassi-Dibai D., Goulart C.D.L., Dixit S., Back G.D., Mendes R.G. (2021). Exercise-Based Rehabilitation Delivery Models in Comorbid Chronic Pulmonary Disease and Chronic Heart Failure. Front. Cardiovasc. Med..

[36] Huang X., Pang S., Zhao Y., Qian J., Zhong J., Liu S. (2023). Efficacy and safety of different traditional Chinese health exercises in patients with coronary heart disease combined with chronic heart failure: A network meta-analysis. Medicine.

[37] Wefer F., Krüger L., Waldréus N. (2024). Non-pharmacological interventions to reduce thirst in patients with heart failure or hemodialysis: A systematic review and meta-analysis. Heart Lung.

[38] Molloy C., Long L., Mordi I.R., Bridges C., Sagar V.A., Davies E.J., Coats A.J., Dalal H., Rees K., Singh S.J. (2024). Exercise-based cardiac rehabilitation for adults with heart failure. Cochrane Libr..

[39] Nick J.M., Roberts L.R., Petersen A.B. (2021). Effectiveness of telemonitoring on self-care behaviors among community-dwelling adults with heart failure: A quantitative systematic review. JBI Evid. Synth..

[40] Dennis S., Kwok W., Alison J., Hassett L., Nisbet G., Refshauge K., Sherrington C., Williams A. (2024). How effective are allied health group interventions for the management of adults with long-term conditions? An umbrella review of systematic reviews and its applicability to the Australian primary health system. BMC Prim. Care.

[41] Peters M.D.J., Marnie C., Tricco A.C., Pollock D., Munn Z., Alexander L., McInerney P., Godfrey C.M., Khalil H. (2020). Updated methodological guidance for the conduct of scoping reviews. JBI Evid. Synth..

[42] Page M.J., McKenzie J.E., Bossuyt P.M., Boutron I., Hoffmann T.C., Mulrow C.D., Shamseer L., Tetzlaff J.M., Akl E.A., Brennan S.E. (2021). The PRISMA 2020 statement: An updated guideline for reporting systematic reviews. BMJ.

[43] Barker T.H., Stone J.C., Sears K., Klugar M., Tufanaru C., Leonardi-Bee J., Aromataris E., Munn Z. (2023). The revised JBI critical appraisal tool for the assessment of risk of bias for randomized controlled trials. JBI Evid. Synth..

[44] Barker T.H., Habibi N., Aromataris E., Stone J.C., Leonardi-Bee J., Sears K., Hasanoff S., Klugar M., Tufanaru C., Moola S. (2024). The revised JBI critical appraisal tool for the assessment of risk of bias for quasi-experimental studies. JBI Evid. Synth..

[45] Lockwood C., Munn Z., Porritt K. (2015). Qualitative research synthesis: Methodological guidance for systematic reviewers utilizing meta-aggregation. Int. J. Evid. Based Healthc..

[46] Dickson V.V., Melkus G.D., Katz S., Levine-Wong A., Dillworth J., Cleland C.M., Riegel B. (2014). Building skill in heart failure self-care among community dwelling older adults: Results of a pilot study. Patient Educ. Couns..

[47] Radhakrishnan K., Toprac P., O’Hair M., Bias R., Kim M.T., Bradley P., Mackert M. (2016). Interactive Digital e-Health Game for Heart Failure Self-Management: A Feasibility Study. Games Health J..

[48] Kalter-Leibovici O., Freimark D., Freedman L.S., Kaufman G., Ziv A., Murad H., Benderly M., Silverman B.G., Friedman N., Cukierman-Yaffe T. (2017). Disease management in the treatment of patients with chronic heart failure who have universal access to health care: A randomized controlled trial. BMC Med..

[49] Antonicelli R., Spazzafumo L., Scalvini S., Olivieri F., Matassini M.V., Parati G., Del Sindaco D., Gallo R., Lattanzio F. (2016). Exercise: A “new drug” for elderly patients with chronic heart failure. Aging.

[50] Yeh G.Y., Chan C.W., Wayne P.M., Conboy L. (2016). The Impact of Tai Chi Exercise on Self-Efficacy, Social Support, and Empowerment in Heart Failure: Insights from a Qualitative Sub-Study from a Randomized Controlled Trial. PLoS ONE.

[51] Dibben G.O., Hillsdon M., Dalal H.M., Tang L.H., Doherty P.J., Taylor R. (2023). Home-based cardiac rehabilitation and physical activity in people with heart failure: A secondary analysis of the REACH-HF randomised controlled trials. BMJ Open.

[52] Gholami M., Zohrabi Salari F., Yarahmadi R., Mokhayeri Y., Veiskaramian A., Amin A. (2024). Effects of balance training on cognitive function and activities of daily living in older adult patients with heart failure: A randomized controlled trial. Ir. J. Med. Sci..

[53] Donesky D., Selman L., McDermott K., Citron T., Howie-Esquivel J. (2017). Evaluation of the Feasibility of a Home-Based TeleYoga Intervention in Participants with Both Chronic Obstructive Pulmonary Disease and Heart Failure. J. Altern. Complement. Med..

[54] Lind L., Carlgren G., Karlsson D. (2016). Old-and with Severe Heart Failure: Telemonitoring by Using Digital Pen Technology in Specialized Homecare: System Description, Implementation, and Early Results. Comput. Inform. Nurs..

[55] Gokalp H., de Folter J., Verma V., Fursse J., Jones R., Clarke M. (2018). Integrated Telehealth and Telecare for Monitoring Frail Elderly with Chronic Disease. Telemed. J. E Health.

[56] Nouryan C.N., Morahan S., Pecinka K., Akerman M., Lesser M., Chaikin D., Castillo S., Zhang M., Pekmezaris R. (2019). Home Telemonitoring of Community-Dwelling Heart Failure Patients After Home Care Discharge. Telemed. J. E Health.

[57] Persson H.L., Lyth J., Lind L. (2020). The Health Diary Telemonitoring and Hospital-Based Home Care Improve Quality of Life Among Elderly Multimorbid COPD and Chronic Heart Failure Subjects. Int. J. Chron. Obstr. Pulmon Dis..

[58] Danduboyina A., Panjiyar B.K., Borra S.R., Panicker S.S. (2023). Cardiovascular Benefits of Resistance Training in Patients with Heart Failure with Reduced Ejection Fraction: A Systematic Review. Cureus.

[59] Durdu H., Demir R., Zeren M., Aydin E., Gunaydin Z.Y., Yigit Z. (2024). The Effect of Computerized Wobble Board and Core Stabilization Exercises on Balance Performance and Exercise Capacity in Patients with Heart Failure: A Randomized Controlled Trial. Arch. Phys. Med. Rehabil..

[60] Hirashiki A., Shimizu A., Kamihara T., Kokubo M., Hashimoto K., Ueda I., Sato K., Kawamura K., Itoh N., Murohara T. (2024). Randomized Controlled Trial of Cardiac Rehabilitation Using the Balance Exercise Assist Robot in Older Adults with Cardiovascular Disease. J. Cardiovasc. Dev. Dis..

[61] Cai Q., Cai S.-B., Chen J.-K., Bai X.-H., Jing C.-X., Zhang X., Li J.-Q. (2022). Tai Chi for Anxiety and Depression Symptoms in Cancer, Stroke, Heart Failure, and Chronic Obstructive Pulmonary Disease: A Systematic Review and Meta-Analysis. Complement Ther. Clin. Pract..

[62] Eimer S., Mahmoodi-Shan G.R., Abdollahi A.A. (2023). The Effect of Self-Care Education on Adherence to Treatment in Elderly Patients with Heart Failure: A Randomized Clinical Trial. Iran. J. Nurs. Midwifery Res..

[63] Ariyanto H., Rosa E.M. (2024). Effectiveness of Telenursing in Improving Quality of Life in Patients with Heart Failure: A Systematic Review and Meta-Analysis. J. Taibah Univ. Med. Sci..

[64] Knox L., Rahman R.J., Beedie C. (2017). Quality of Life in Patients Receiving Telemedicine Enhanced Chronic Heart Failure Disease Management: A Meta-Analysis. J. Telemed. Telecare.

[65] Roaquin L., Apsay K.L., Pangan C.R., Hangdaan L., Lin Y. (2025). Telemonitoring in Chronic Heart Failure Among the Elderly: A Rapid Review of Literature. SAGE Open Nurs..

[66] Seto E., Morita P.P., Tomkun J., Lee T.M., Ross H., Reid-Haughian C., Kaboff A., Mulholland D., Cafazzo J.A. (2019). Implementation of a Heart Failure Telemonitoring System in Home Care Nursing: Feasibility Study. JMIR Med. Inform..

[67] Umeh C.A., Torbela A., Saigal S., Kaur H., Kazourra S., Gupta R., Shah S. (2022). Telemonitoring in heart failure patients: Systematic review and meta-analysis of randomized controlled trials. World J. Cardiol..

[68] Yamamoto S., Okamura M., Akashi Y.J., Tanaka S., Shimizu M., Tsuchikawa Y., Ashikaga K., Kamiya K., Kato Y., Nakayama A. (2024). Impact of Long-Term Exercise-Based Cardiac Rehabilitation in Patients with Chronic Heart Failure—A Systematic Review and Meta-Analysis. Circ. J..

[69] Ferreira J.P., Metra M., Anker S.D., Dickstein K., Lang C.C., Ng L., Samani N.J., Cleland J.G., van Veldhuisen D.J., Voors A.A. (2019). Clinical correlates and outcome associated with changes in 6-minute walking distance in patients with heart failure: Findings from the BIOSTAT-CHF study. Eur. J. Heart Fail..

[70] Kucio C., Stastny P., Leszczyńska-Bolewska B., Engelmann M., Kucio E., Uhlir P., Stania M., Polak A. (2018). Exercise-Based Cardiac Rehabilitation with and Without Neuromuscular Electrical Stimulation and its Effect on Exercise Tolerance and Life Quality of Persons with Chronic Heart Failure. J. Hum. Kinet..

[71] Abdelbasset W.K., Alqahtani B.A., Elshehawy A.A., Tantawy S.A., Elnegamy T.E., Kamel D.M. (2019). Examining the impacts of 12 weeks of low to moderate-intensity aerobic exercise on depression status in patients with systolic congestive heart failure—A randomized controlled study. Clinics.

[72] Schaich C.L., Hughes T.M., Kitzman D.W., Jung Y., Chen H., Nicklas B.J., Houston D.K., Brubaker P.H., Molina A.J.A., Hugenschmidt C.E. (2024). Neurocognitive Impairments and Their Improvement Following Exercise and Dietary Interventions in Older Patients with Heart Failure with Preserved Ejection Fraction. Circ. Heart Fail..

[73] Bone J.K., Bu F., Fluharty M.E., Paul E., Sonke J.K., Fancourt D. (2022). Engagement in leisure activities and depression in older adults in the United States: Longitudinal evidence from the Health and Retirement Study. Soc. Sci. Med..

[74] Yang C., Lee D.T.F., Wang X., Chair S.Y. (2022). Effects of a nurse-led medication self-management intervention on medication adherence and health outcomes in older people with multimorbidity: A randomised controlled trial. Int. J. Nurs. Stud..

[75] Hernández Pinzón C., Flórez-Flórez M. (2016). Adherencia al tratamiento en la insuficiencia cardiaca y las tecnologías de la información y la comunicación. Rev. Colomb. Cardiol..

[76] Masotta V., Dante A., Caponnetto V., Marcotullio A., Ferraiuolo F., Bertocchi L., Camero F., Lancia L., Petrucci C. (2024). Telehealth care and remote monitoring strategies in heart failure patients: A systematic review and meta-analysis. Heart Lung.

[77] Camino Ortega E., Baroja Gil de Gómez A., González Gamarra A., Cuevas-Budhart M.A., García Klepzig J.L., Gómez del Pulgar García-Madrid M. (2023). Educación terapéutica en insuficiencia cardiaca mediante e-Salud: Revisión sistemática. Aten. Prim..

[78] Dugal J.K., Malhi A.S., Ramazani N., Yee B., DiCaro M.V., Lei K. (2024). Non-Pharmacological Therapy in Heart Failure and Management of Heart Failure in Special Populations—A Review. J. Clin. Med..

[79] Abbasi-Ghahramanloo A., Soltani-Kermanshahi M., Mansori K., Khazaei-Pool M., Sohrabi M., Baradaran H.R., Talebloo Z., Gholami A. (2020). Comparison of SF-36 and WHOQoL-BREF in Measuring Quality of Life in Patients with Type 2 Diabetes. Int. J. Gen. Med..

[80] González-Guerrero J.L., Hernández-Mocholi M.A., Ribera-Casado J.M., García-Mayolín N., Alonso-Fernández T., Gusi N. (2018). Cost-effectiveness of a follow-up program for older patients with heart failure: A randomized controlled trial. Eur. Geriatr. Med..

[81] Scholte N.T.B., Gürgöze M.T., Aydin D., Theuns D.A.M.J., Manintveld O.C., Ronner E., Boersma E., de Boer R.A., van der Boon R.M.A., Brugts J.J. (2023). Telemonitoring for heart failure: A meta-analysis. Eur. Heart J..

[82] Checa C., Canelo-Aybar C., Suclupe S., Ginesta-López D., Berenguera A., Castells X., Brotons C., Posso M. (2022). Effectiveness and Cost-Effectiveness of Case Management in Advanced Heart Failure Patients Attended in Primary Care: A Systematic Review and Meta-Analysis. Int. J. Environ. Res. Public Health.

[83] Driscoll A., Gao L., Watts J.J. (2022). Clinical effectiveness and cost-effectiveness of ambulatory heart failure nurse-led services: An integrated review. BMC Cardiovasc. Disord..

[84] Amasene M., Besga A., Echeverria I., Urquiza M., Ruiz J.R., Rodriguez-Larrad A., Aldamiz M., Anaut P., Irazusta J., Labayen I. (2019). Effects of Leucine-Enriched Whey Protein Supplementation on Physical Function in Post-Hospitalized Older Adults Participating in 12-Weeks of Resistance Training Program: A Randomized Controlled Trial. Nutrients.

[85] Paneroni M., Scalvini S., Corrà U., Lovagnini M., Maestri R., Mazza A., Raimondo R., Agostoni P., La Rovere M.T. (2022). The Impact of Cardiac Rehabilitation on Activities of Daily Life in Elderly Patients with Heart Failure. Front. Physiol..

[86] Siddiqi A.K., Shahzad M., Kumar A., Ahmed M., Sridharan L., Abdou M.H., Naeem M. (2025). The efficacy of inspiratory muscle training in improving clinical outcomes in heart failure patients: An updated systematic review and meta-analysis. J. Cardiol..

[87] Hui J., Wang Y., Zhao J., Cong W., Xu F. (2022). Effects of Tai Chi on health status in adults with chronic heart failure: A systematic review and meta-analysis. Front. Cardiovasc. Med..

[88] Shin S. (2021). Meta-Analysis of the Effect of Yoga Practice on Physical Fitness in the Elderly. Int. J. Environ. Res. Public Health.

[89] Zhao Q., Liu X., Wan X., Yu X., Cao X., Yang F., Cai Y. (2023). Non-pharmacological interventions for cognitive impairment in older adults with heart failure: A systematic review. Geriatr. Nurs..

